# Daprodustat Accelerates High Phosphate-Induced Calcification Through the Activation of HIF-1 Signaling

**DOI:** 10.3389/fphar.2022.798053

**Published:** 2022-02-07

**Authors:** Andrea Tóth, Dávid Máté Csiki, Béla Nagy, Enikő Balogh, Gréta Lente, Haneen Ababneh, Árpád Szöőr, Viktória Jeney

**Affiliations:** ^1^ MTA-DE Lendület Vascular Pathophysiology Research Group, Research Centre for Molecular Medicine, Faculty of Medicine, University of Debrecen, Debrecen, Hungary; ^2^ Doctoral School of Molecular Cell and Immune Biology, Faculty of Medicine, University of Debrecen, Debrecen, Hungary; ^3^ Department of Laboratory Medicine, Faculty of Medicine, University of Debrecen, Debrecen, Hungary; ^4^ Department of Biophysics and Cell Biology, Faculty of Medicine, University of Debrecen, Debrecen, Hungary

**Keywords:** chronic kidney disease (CKD), vascular calcification, anemia, hypoxia-inducible factor-1, prolyl hydroxylase inhibitor (PHI), daprodustat, vascular smooth muscle cell

## Abstract

**Aims:** Chronic kidney disease (CKD) is frequently associated with other chronic diseases including anemia. Daprodustat (DPD) is a prolyl hydroxylase inhibitor, a member of a family of those new generation drugs that increase erythropoiesis *via* activation of the hypoxia-inducible factor 1 (HIF-1) pathway. Previous studies showed that HIF-1 activation is ultimately linked to acceleration of vascular calcification. We aimed to investigate the effect of DPD on high phosphate-induced calcification.

**Methods and Results:** We investigated the effect of DPD on calcification in primary human aortic vascular smooth muscle cells (VSMCs), in mouse aorta rings, and an adenine and high phosphate-induced CKD murine model. DPD stabilized HIF-1α and HIF-2α and activated the HIF-1 pathway in VSMCs. Treatment with DPD increased phosphate-induced calcification in cultured VSMCs and murine aorta rings. Oral administration of DPD to adenine and high phosphate-induced CKD mice corrected anemia but increased aortic calcification as assessed by osteosense staining. The inhibition of the transcriptional activity of HIF-1 by chetomin or silencing of HIF-1α attenuated the effect of DPD on VSMC calcification.

**Conclusion:** Clinical studies with a long follow-up period are needed to evaluate the possible risk of sustained activation of HIF-1 by DPD in accelerating medial calcification in CKD patients with hyperphosphatemia.

## Introduction

Chronic kidney disease (CKD) is an irreversible and progressive disease associated with alteration of the renal structure and decline of kidney functions (Webster et al., 2017). CKD is a public health problem worldwide affecting about 10% of the general population in high- and middle-income countries (Webster et al., 2017). CKD is frequently associated with other chronic diseases, including anemia (Babitt and Lin, 2012), metabolic bone diseases (Martin and González, 2007), and cardiovascular diseases (Sarnak et al., 2003; Di Angelantonio et al., 2007). CKD patients have five to ten times higher risk of premature death than the general population, which is largely attributed to death from cardiovascular diseases (Sarnak et al., 2003; Di Angelantonio et al., 2007; Webster et al., 2017).

CKD-associated anemia is a considerable burden because it significantly worsens the quality of life of CKD patients, increases hospitalization, causes cognitive impairment, propagates the progression of CKD, and increases the risk of cardiovascular events and mortality. The etiology of CKD-associated anemia is complex with the contribution of reduced production of erythropoietin (EPO), a kidney-derived factor responsible for stimulating erythropoiesis, shortened red blood cell lifespan, and iron deficiency (Babitt and Lin, 2012; Hanna et al., 2021). Accordingly, anemia in patients with advanced CKD was targeted with EPO or erythropoiesis-stimulating agents (ESAs) (Eschbach et al., 1987), along with oral or intravenous iron supplementation (Gafter-Gvili et al., 2019; Batchelor et al., 2020). Red blood cell transfusion remained a treatment option only for blood loss or severe hyporesponsiveness for ESAs (Locatelli et al., 2013).

The treatment of CKD-associated anemia was revolutionized by the introduction of EPO and ESAs, but safety concerns of ESA use have lately been emerged (Robles, 2016). Trials showed that ESA treatment increases the risks for cardiovascular events and probably increases risk for death, serious cardiovascular events, and the development of end-stage renal disease (Palmer et al., 2010; Koulouridis et al., 2013; McCullough et al., 2013). Consequently, following the U.S. Food and Drug Administration (FDA) warning, the use of ESAs has markedly decreased, even in patients with very low hemoglobin levels (<10 mg/dl), and currently, the administration of ESAs is recommended only to avoid red blood cell transfusion.

An alternative therapeutic strategy has emerged to treat CKD-associated anemia that relies on the modulation of the hypoxia-inducible factor (HIF) pathway (Locatelli et al., 2017). The activation of the HIF pathway leads to transcriptional activation of numerous genes, including EPO, and a subsequent increase in erythropoiesis (Semenza and Wang, 1992). Therefore, small-molecule stabilizers of the HIF pathway have been developed (Maxwell and Eckardt, 2016). These molecules inhibit the activity of HIF prolyl hydroxylase domain–containing (PHD) enzymes, which are responsible for hydroxylation of the oxygen-sensitive alpha subunits of HIF at conserved proline residues under normoxic conditions (Semenza, 2001). Proline-hydroxylated α subunits are recognized and ubiquitinated by the von Hippel–Lindau E3 ubiquitin ligase, followed by rapid proteasomal degradation (Jaakkola et al., 2001; Metzen and Ratcliffe, 2004). PHD inhibitors (PHIs) mimic the effect of hypoxia and result in stabilization of the HIF α subunits, nuclear translocation, assembly of the HIF transcription complex, and eventually HIF activation with increased production of EPO.

Morbidity and mortality of CKD patients are largely associated with vascular calcification, an actively regulated process in which vascular smooth muscle cells (VSMCs) undergo an osteochondrogenic transdifferentiation process (Jono et al., 2000; Schoppet et al., 2008; Giachelli, 2009; Paloian and Giachelli, 2014). Various inhibitors and inducers of vascular calcification have been identified, and recent studies highlighted the potential role of hypoxia and the HIF-1 pathway activation in vascular calcification (Mokas et al., 2016; Balogh et al., 2019). Because 1) PHIs target the HIF pathway and 2) hypoxia-mediated activation of HIF-1 induces the calcification of VSMCs, here, we investigated the effect of the PHI daprodustat (GSK1278863, DPD) on calcification in both *in vitro* and *in vivo* conditions.

## Materials and Methods

### Materials

All the reagents were purchased from Sigma-Aldrich Co. (St. Louis, MO, United States) unless otherwise specified.

### Cell Culture and Treatments

Human aortic VSMCs (354-05; Cell Applications Inc., San Diego, CA, United States) were maintained in a growth medium (GM) that was prepared by supplementing Dulbecco’s modified Eagle medium (DMEM, D6171, Sigma) with 10% FBS (10270-106, Gibco, Grand Island, NY, United States), antibiotic–antimycotic solution (A5955, Sigma), sodium pyruvate (S8636, Sigma), and L-glutamine (G7513, Sigma). Cells were maintained at 37°C in a humidified atmosphere containing 5% CO_2_. Cells were grown till they reach confluence and used from passages 5 to 8. To induce calcification, we cultured VSMCs in an osteogenic medium (OM) that was obtained by supplementing GM with different concentrations of inorganic phosphate (Pi) (NaH_2_PO_4_-Na_2_HPO_4_, 1–2.5 mmol/L, pH 7.4). DPD (HY-17608, MedChemExpress, NJ, United States) was dissolved in dimethyl sulfoxide (DMSO, D2438, Sigma) to make a stock solution (25 mmol/L) and used in concentrations between 1 and 100 μmol/L. A hypoxic condition was obtained by placing the cells into a modular incubator chamber (Billups-Rothenburg Inc. Del Mar, CA, United States), which was connected to a gas bottle containing a mixture of 1% O_2_, 5% CO_2_, and 94% of N_2_ (Messer Group GmbH, Bad Soden, Germany). A continuous slow flow (0.1 L/min) was applied throughout the experiment. In some experiments, we used chetomin (stock solution: 12.5 μmol/L in DMSO; working concentration: 12.5 nmol/L; C8106, Sigma) to inhibit the HIF-1 signaling pathway. Uric acid stock solution (80 mmol/L) was prepared in 1 mmol/L NaOH.

### Alizarin Red (AR) Staining and Quantification

After washing with DPBS, the cells were fixed in 4% paraformaldehyde (16005, Sigma) and rinsed with deionized water thoroughly. Cells were stained with Alizarin Red S (A5533, Sigma) solution (2%, pH 4.2) for 20 min at room temperature. Excessive dye was removed by several washes in deionized water. To quantify AR staining in 96-well plates, we added 100 μL of hexadecylpyridinium chloride (C9002, Sigma) solution (100 mmol/L) to the wells and measured optical density (OD) at 560 nm using hexadecylpyridinium chloride solution as blank.

### Quantification of Ca Deposition

Cells grown on 96-well plates were washed twice with DPBS and decalcified with HCl (30721, Sigma, 0.6 mol/L) for 30 min at room temperature. The Ca content of the HCl supernatants was determined by using a QuantiChrome Calcium Assay Kit (DICA-500, Gentaur, Kampenhout, Belgium). Following decalcification, cells were washed twice with DPBS and solubilized with a solution of NaOH (S8045, Sigma, 0.1 mol/L) and sodium dodecyl sulfate (11667289001, Sigma, 0.1%), and protein content of samples were measured using the BCA protein assay kit (23225, Pierce Biotechnology, Rockford, IL, United States). The Ca content of the cells was normalized to protein content and expressed as mg/mg protein. The observer who performed all the Ca measurements was blinded to the group assignment.

### Quantification of OCN and VEGF-A

For OCN detection, the ECM of the cells grown on 6-well plates was dissolved in 100 μL of EDTA (E6758, Sigma, 0.5 mol/L, pH 6.9). OCN content of the EDTA-solubilized ECM samples was quantified by an enzyme-linked immunosorbent assay (ELISA) (DY1419-05, DuoSet ELISA, R&D, Minneapolis, MN, United States), according to manufacturer’s protocol. VEGF-A levels were quantified from the cellular supernatant using a VEGF-A ELISA kit (DY293B-05, DuoSet ELISA, R&D, Minneapolis, MN, United States). The observer who performed all the ELISA measurements was blinded to the group assignment.

### *Ex Vivo* Aorta Organ Culture Model and Quantification of Aortic Calcium

C57BL/6 mice (8- to 12-week-old male, *n* = 18) were exterminated by CO_2_ inhalation and perfused with 5 ml of sterile DPBS. The entire aorta was harvested and cleaned under aseptic conditions and cut into pieces. Aorta rings were randomly divided into three groups and maintained in control, high Pi (2 mmol/L Pi), and high Pi (2 mmol/L Pi) plus DPD (25 μmol/L) conditions, respectively. The culturing medium was DMEM (D6171, Sigma) supplemented with 10% FBS (10,270-106, Gibco, Grand Island, NY, United States), antibiotic–antimycotic solution (A5955, Sigma), sodium pyruvate (S8636, Sigma), l-glutamine (G7513, Sigma), and 2.5 μg/ml amphotericin B (171,375, Millipore). The medium was changed every 2 days. After the 3rd, 5th, and 7th day, the aorta pieces were washed in DPBS, opened longitudinally and decalcified in 25 µL of 0.6 mmol/L HCl overnight. The Ca content was determined by using the QuantiChrom Ca-assay kit, as described earlier. The observer who performed aorta Ca measurements was blinded to the group assignment.

### CKD Induction and DPD Treatment in Mice

Animal care and experimental procedures were performed in accordance with the institutional and national guidelines and was approved by the Institutional Ethics Committee of University of Debrecen (registration number 3/2018/DEMÁB). Animal studies were reported in compliance with the ARRIVE guidelines. Male C57BL/6 mice (8- to 12-week-old, *n* = 25) were randomly divided into 5 groups, control (Ctrl), CKD, CKD + DPD (5 mg/kg/day), CKD + DPD (10 mg/kg/day), and CKD + DPD (15 mg/kg/day). CKD was induced by a two-phase diet as described previously (Tani et al., 2017). In the first 6 weeks, the mice received a diet containing 0.2% adenine and 0.7% phosphate, followed by a diet containing 0.2% adenine and 1.8% phosphate (S8106-S075 and S8893-S006, respectively; Ssniff, Soest, Germany) for 3 weeks. Mice were housed in cages with standard beddings and unlimited access to food and water. DPD (HY-17608, MedChemExpress, NJ, United States) was suspended in 1% methylcellulose and was daily administered orally at a dose of 5/10/15 mg/kg between weeks 7 and 9. At the end of the experiment mice were euthanized by CO_2_ inhalation, blood was collected by cardiac puncture and aortas were harvested for calcium analysis and histology. In a separate experiment, C57BL/6 mice (8- to 12-week-old male, *n* = 15) were randomly divided into three groups: Ctrl, CKD, and CKD + DPD (15 mg/kg/day). The experiment was performed as the previous one, and aorta calcification was assessed by near-infrared imaging (detailed separately).

### Laboratory Analysis of Renal Function and Anemia in CKD Mice

Serum phosphorous, urea creatinine, and uric acid levels were determined in mice by kinetic assays on a Cobas^®^ c502 instrument (Roche Diagnostics, Mannheim, Germany). K_3_-EDTA anticoagulated whole blood murine samples were analyzed by a Siemens Advia 2120i hematology analyzer (Tarrytown, NY, United States) with the 800 Mouse C57BL program of Multispecies software. Hemoglobin concentration was measured by using a cyanide-free colorimetric method. Hematocrit values were determined as a calculated parameter derived from the red blood cell count (RBC in T/L) and mean cell volume (MCV in fL). The number of RBCs was multiplied by the MCV of the sample RBCs and was divided by 1,000. The observer who performed these measurements was blinded to the group assignment.

### Near-Infrared Imaging and Quantification of Aortic Calcification

OsteoSense dye (OsteoSense 680 EX and NEV10020EX; PerkinElmer, MA, United States) and near-infrared imaging were used to evaluate aorta calcification in mice as previously described (Malhotra et al., 2019). Mice (Ctrl, CKD, and CKD + DPD; *n* = 5/group) were anesthetized with isoflurane and injected with 2 nmol OsteoSense dye dissolved in 100 µL DPBS retro-orbitally. After 24 hours, mice were killed by CO_2_ inhalation; the mice were perfused with 5 ml of ice-cold PBS, and aortas were isolated, cleaned, and analyzed *ex vivo* by an IVIS Spectrum *In Vivo* Imaging System (PerkinElmer, MA, United States). The observer who performed this measurement was blinded to the group assignment.

### Histology

Aortic rings were fixed in 10% neutral-buffered formalin (HT501640; Sigma), embedded in paraffin blocks, and cut into 4-µm-thick cross sections. After deparaffinization and rehydration, we performed von Kossa staining and hematoxylin eosin (H&E) counterstaining on the sections, according to manufacturer’s protocol (von Kossa Kit, ab150687; Abcam, Cambridge, United Kingdom). The observer who performed histology was blinded to the group assignment.

### Western Blot Analysis

VSMCs were lysed in the Laemmli lysis buffer (38,733, Sigma). Proteins were resolved by SDS-PAGE (7.5 and 10%) and transferred to nitrocellulose membranes (1060003; Amersham, GE Healthcare, Chicago, IL, United States). Western blotting was performed by using anti-HIF1α antibody (GTX127309, GeneTex, Irvine, CA, United States) at 0.5 μg/ml concentration, anti-HIF2α antibody (#7096, Cell Signaling Technology, Danvers, MA, United States) at 2.5 μg/ml concentration, and anti-Glut-1 antibody (GTX1309, GeneTex) at 0.5 μg/ml concentration. After binding of the primary antibodies, membranes were incubated with horseradish peroxidase–linked rabbit (NA-934) and mouse IgG (NA-931) (Amersham, GE Healthcare) at 0.5 μg/ml concentration. Antigen–antibody complexes were visualized using the enhanced chemiluminescence system Clarity Western ECL (170-5060, BioRad, Hercules, CA, United States). Chemiluminescent signals were detected conventionally on an X-ray film or digitally by using a C-Digit Blot Scanner (LI-COR Biosciences, Lincoln, NE, United States). After detection, the membranes were stripped and reprobed for β-actin using an anti-β-actin antibody (sc-47778, Santa Cruz Biotechnology Inc., Dallas, TX, United States) at 0.2 μg/ml concentration. Western blots were repeated three times with independent sample sets and blots were quantified by using the inbuilt software on the C-Digit Blot Scanner.

### RNA Silencing

To knock-down HIF-1α gene expression, we used *Silencer* ^®^ select siRNA construct targeting HIF-1α (assay IDs #AM16708, Thermo Fisher Scientific). As a control, we used negative control #1 construct (#4390843, Thermo Fisher Scientific). The Lipofectamine^®^ RNAiMAX reagent (13778075, Invitrogen, Carlsbad, CA, United States) was used to transfect VSMCs, according to manufacturer’s protocol.

### Statistical Analysis

Group size was equal in all experiments, and no data points were excluded from the analysis. Data are presented as mean ±SD with individual data points. Statistical analyses were performed with GraphPad Prism software (version 8.01, San Diego, CA, United States). Comparisons between more than two groups were carried out by one-way ANOVA, followed by Tukey’s multiple comparisons test. To compare each treatment group with a single control group, we performed one-way ANOVA, followed by Dunnett’s *post hoc* test. Time course experiments were analyzed by two-way ANOVA, followed by Tukey’s multiple comparisons test. The value of *p* < 0.05 was considered significant.

## Results

### Hypoxia Accelerates Pi-Induced ECM Calcification in VSMCs

**FIGURE 1:**
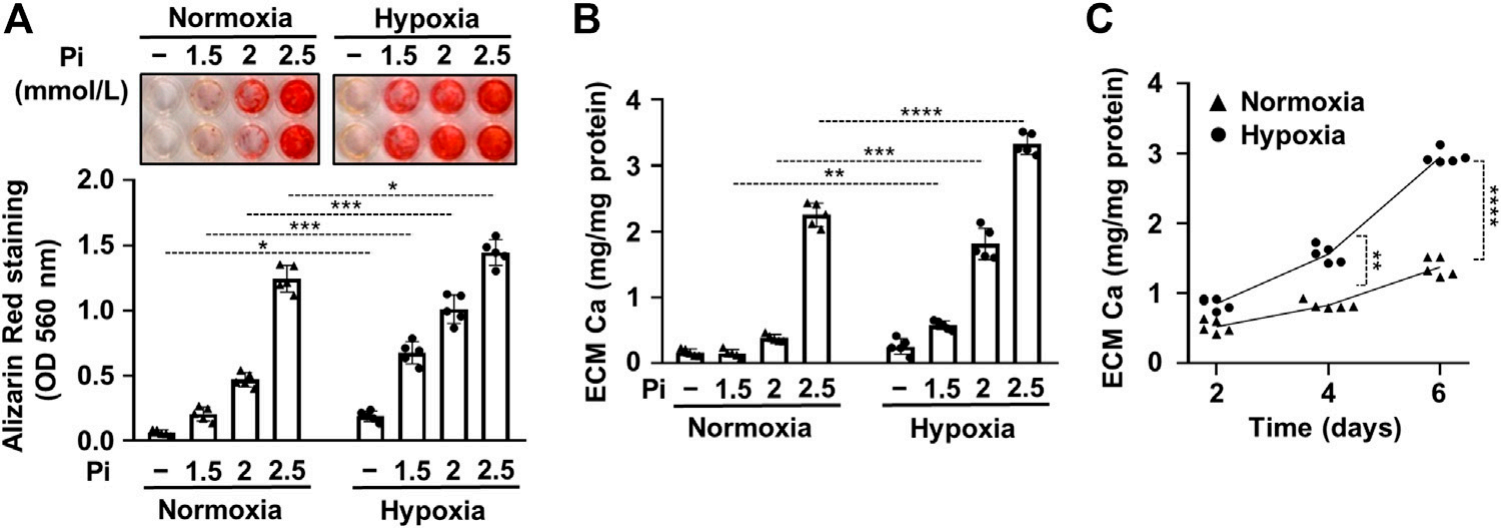
Hypoxia increases Pi-mediated extracellular matrix calcification of VSMCs. Confluent VSMCs were exposed to a calcification medium containing Pi (1.5–2.5 mmol/L) under normoxic (21% O_2_) and hypoxic conditions (1% O_2_). **(A)** Ca deposition in the ECM (day 6) evaluated by AR staining. Representative image and quantification are depicted. **(B)** Ca content of the HCl-solubilized ECM. **(C)** Time course of Ca accumulation under normoxic and hypoxic conditions in the presence of 2.5 mmol/L Pi. **(A–C)** Data are expressed as mean ± SD, *n* = 5. **(A,B)** Ordinary one-way ANOVA followed by Tukey’s multiple comparisons test were used to obtain *p* values. **(C)** Multiple t tests to compare normoxia and hypoxia samples at each time points were performed to obtain *p* values. *p < 0.05, **p < 0.01, ***p < 0.005, ****p < 0.001.

A previous study by Mokas et al. showed that hypoxia amplifies the pro-calcification effect of elevated inorganic phosphate (Pi) (Mokas et al., 2016). To confirm this finding, first, we set up an *in vitro* model of vascular calcification with cultured human VSMCs maintained in a calcification medium that was supplemented with different concentrations of Pi (0–2.5 mmol/L) under normoxic (21% O_2_) and hypoxic (1% O_2_) conditions. Calcification was evaluated by AR staining after 6 days of treatment (Figure 1A). We found increased intensity of AR staining at all tested Pi concentrations under hypoxia in comparison to normoxic condition (Figure 1A). Interestingly, hypoxia increased AR staining intensity over normoxia control after 6 days of treatment even in normal phosphate condition. Measurement of Ca levels confirmed the result of AR staining, having more Ca in the extracellular matrix (ECM) of VSMCs under hypoxic condition than normoxic groups (Figure 1B). In a time course experiment, we investigated the kinetics of calcification in the presence of a calcification medium containing 2.5 mmol/L excess Pi under normoxic and hypoxic conditions (Figure 1C). Results show that calcification is significantly higher on both days 4 and 6 under hypoxia than under normoxia (Figure 1C). These results confirm the previously established pro-calcifying effect of hypoxia under normal and high Pi conditions (Mokas et al., 2016; Balogh et al., 2019).

### DPD Activates the HIF-1 Pathway and Increases Pi-Induced Calcification in VSMCs *In Vitro* and Aorta *Ex Vivo*

**FIGURE 2:**
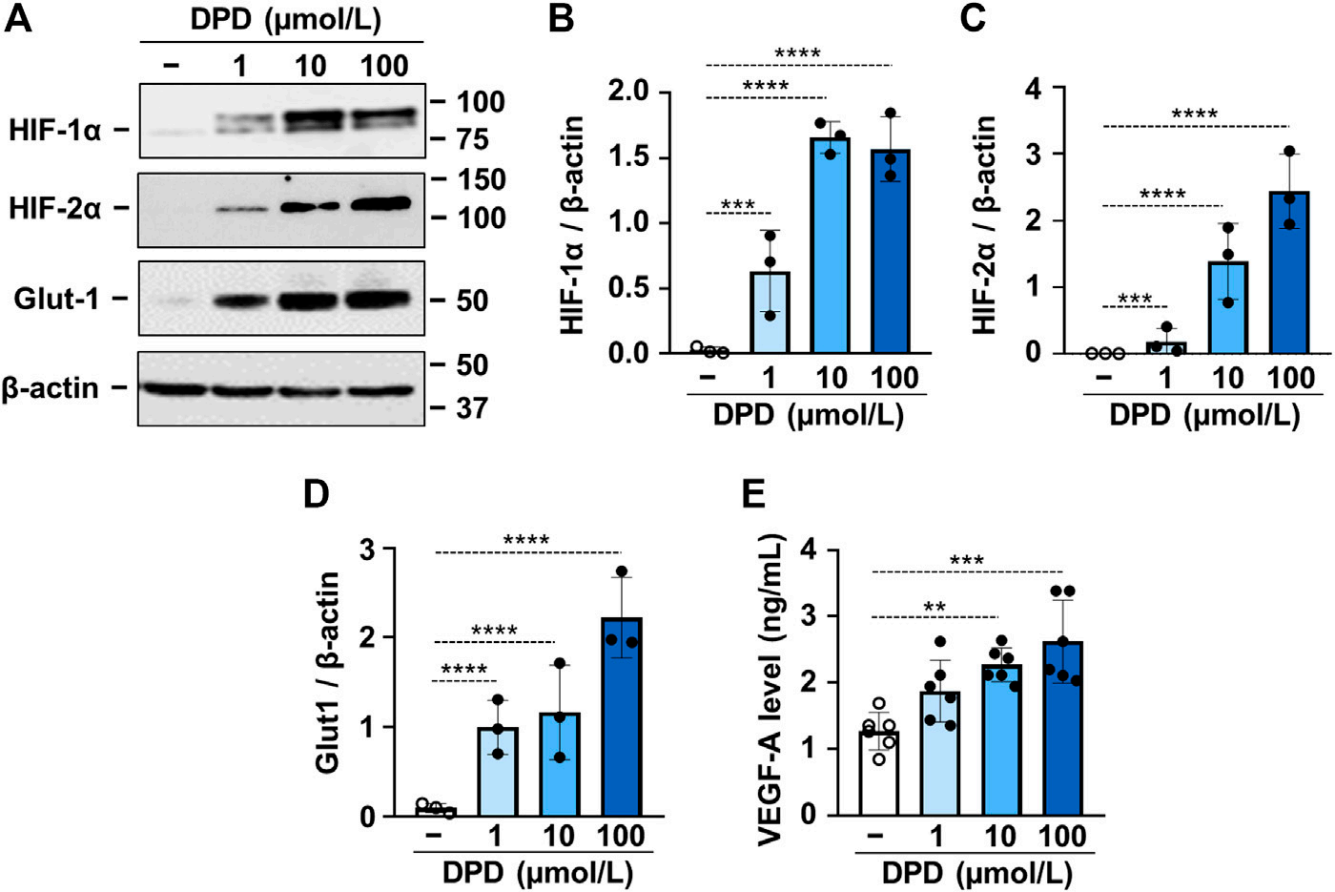
DPD triggers hypoxia response in VSMCs. VSMCs were cultured in the absence or presence of DPD (1–100 μmol/L). **(A–D)** Protein expression of HIF-1α, HIF-2α, and Glut-1 in whole cell lysates was evaluated after 24 h. Membranes were reprobed for β-actin. **(A)** Representative Western blots and densitometry analyses on the relative expression of **(B)** HIF-1α, **(C)** HIF-2α, and **(D)** Glut-1 normalized to β-actin are depicted (*n* = 3). **(E)** VEGF-A levels (24 h) were determined from cellular supernatant by ELISA (*n* = 6). Data are expressed as mean ± SD. **(A–D)** Ordinary one-way ANOVA followed by Dunnett’s multiple comparisons test and **(E)** ordinary one-way ANOVA followed by Tukey’s multiple comparisons test were used to obtain *p* values. **p < 0.01, ***p < 0.005, ****p < 0.001.

DPD is a prolyl hydroxylase inhibitor; therefore, next, we investigated its effect on HIF-1 activation in VSMCs. We found that DPD (1–100 μmol/L) increased the expression of both HIF-1α and HIF-2α in a dose-dependent manner (Figures 2A–C). This was accompanied by increased expression of glucose transporter 1 (Glut-1) and vascular endothelial growth factor A (VEGF-A), which are characteristic target genes of the HIF-1 pathway (Figures 2A,D,E).

**FIGURE 3:**
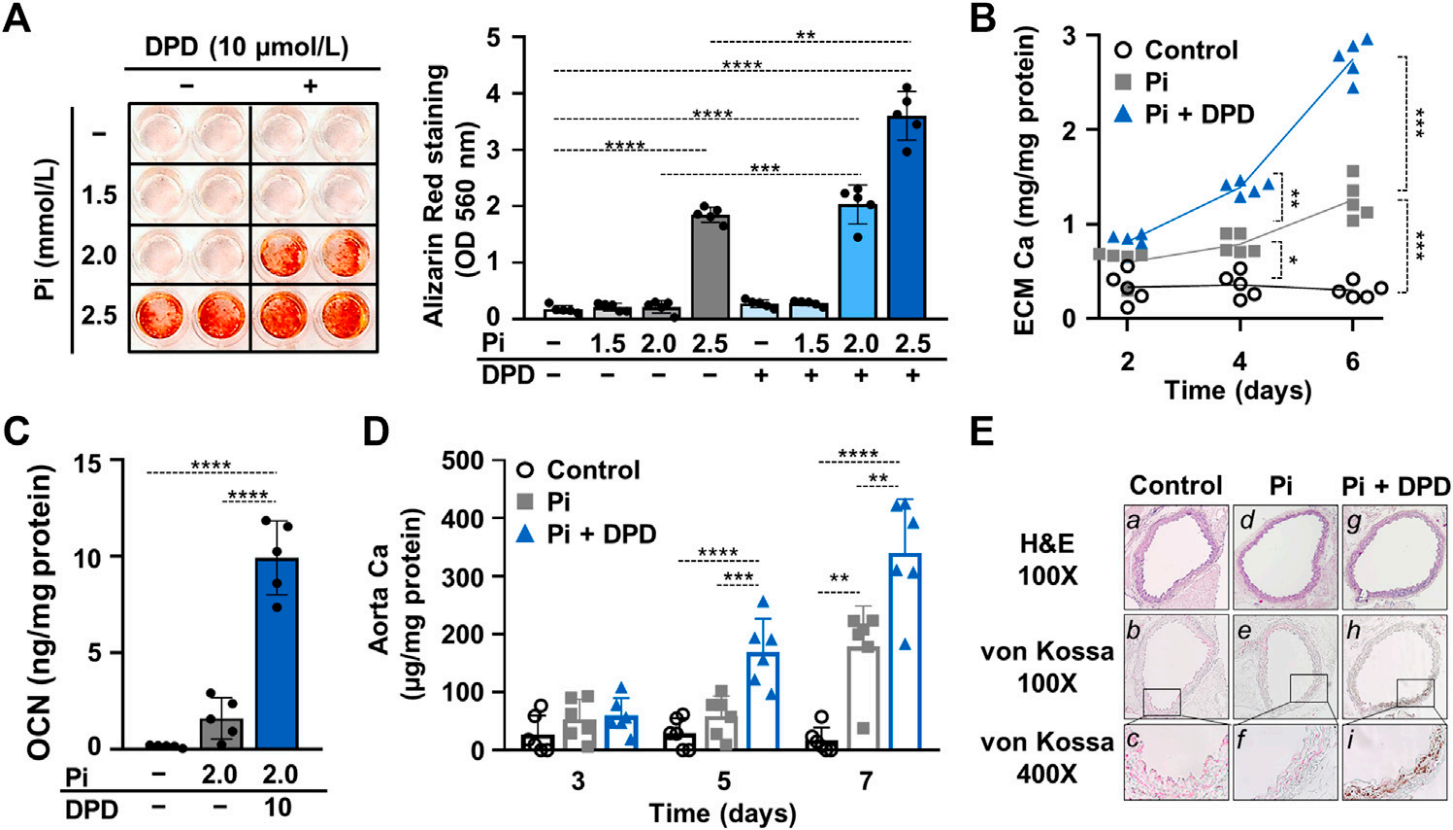
DPD increases Pi-mediated ECM calcification and osteochondrogenic transdifferentiation of VSMCs and accelerates aorta ring calcification. **(A–C)** VSMCs were cultured in an osteogenic medium (excess Pi: 1.5–2.5 mmol/L) in the presence or absence of DPD (10 μmol/L). **(A)** Representative AR staining (day 6) and quantification. **(B)** Time course of Ca accumulation induced by Pi (2 mmol/L) and Pi + DPD (10 μmol/L) (day 2, 4, and 6). **(C)** OCN level (day 6) in EDTA-solubilized ECM samples. **(D)** Aortic rings obtained from C57BL/6 mice were cultured in control, high Pi (2 mmol/L), and high Pi + DPD (25 μmol/L) conditions. Ca content of aorta rings normalized to protein level (day 3, day 5, and day 7). **(E)** Histological analysis of *ex vivo* cultured aortic rings from C57BL/6 mice. Representative H&E and von Kossa-stained aortic sections of untreated, Pi-, and Pi + DPD-treated aorta rings (day 7). Magnification: ×100 and ×400. Data are expressed as mean ± SD, *n* = 5. **(A,C)** Ordinary one-way ANOVA followed by Tukey’s multiple comparisons test and **(B,D)** two-way ANOVA followed by Tukey’s multiple comparisons test were used to obtain *p* values. *p < 0.05, **p < 0.01, ***p < 0.005, ****p < 0.001.

Then we addressed the effect of DPD on Pi-induced calcification of VSMCs. We treated VSMCs in the osteogenic medium supplemented with various concentrations of Pi (1.5–2.5 mmol/L) in the presence or absence of DPD (10 μmol/L). Calcification was assessed by AR staining after 6 days of exposure. The osteogenic medium supplemented with 2.0 mmol/L Pi triggered calcification only in the presence of DPD. Higher Pi (2.5 mmol/L) induced calcification in both the absence and presence of DPD, but the extent of calcification was higher in the DPD-treated cells (Figure 3A). Next, we performed a time course experiment and measured the Ca content of the extracellular matrix after 2nd, 4th, and 6 th days of exposure to high Pi (2 mmol/L) in the absence or presence of DPD (Figure 3B). DPD significantly increased Ca content of the ECM on days 4 and 6 (Figure 3B). Next, we investigated the effect of DPD on the expression of osteocalcin (OCN), a major non-collagenous protein of the bone matrix, and an established marker of osteochondrogenic transdifferentiation of VSMCs. We found that DPD (10 μmol/L) largely enhanced Pi-induced increase in OCN production in VSMCs (*p* < 0.0001, Figure 3C). For further confirmation, we set up an *ex vivo* tissue culture model and investigated the effect of DPD on aorta calcification. We cultured cleaned aorta pieces of C57BL/6 mice under control, high Pi (2 mmol/L), and high Pi + DPD (25 μmol/L) conditions and measured Ca levels of aorta rings on day 3, day 5, and day 7. High Pi increased Ca content of the aorta on day 7, whereas when high Pi and DPD were applied together, aorta calcification started already on day 5 (Figure 3D). The calcium content was higher in the aorta rings treated with Pi + DPD than in Pi-treated aorta rings on day 7 (345.9 ± 102.7 vs. 178.3 ± 70.7 μg/mg protein, Figure 3D). After 7 days of treatment, we performed von Kossa staining on the specimens of the descending thoracic aorta to visualize calcification. von Kossa staining revealed calcification in the media layer of the aorta that was treated with Pi + DPD but not in control or Pi-treated aorta specimens (Figure 3E). These results suggest that DPD is a pro-calcification agent that increases Pi-induced calcification of VSMCs and mouse aorta in a synergistic way.

### DPD Administration Successfully Corrects Anemia but Increases Aorta Calcification in a Mice Model of CKD

**FIGURE 4:**
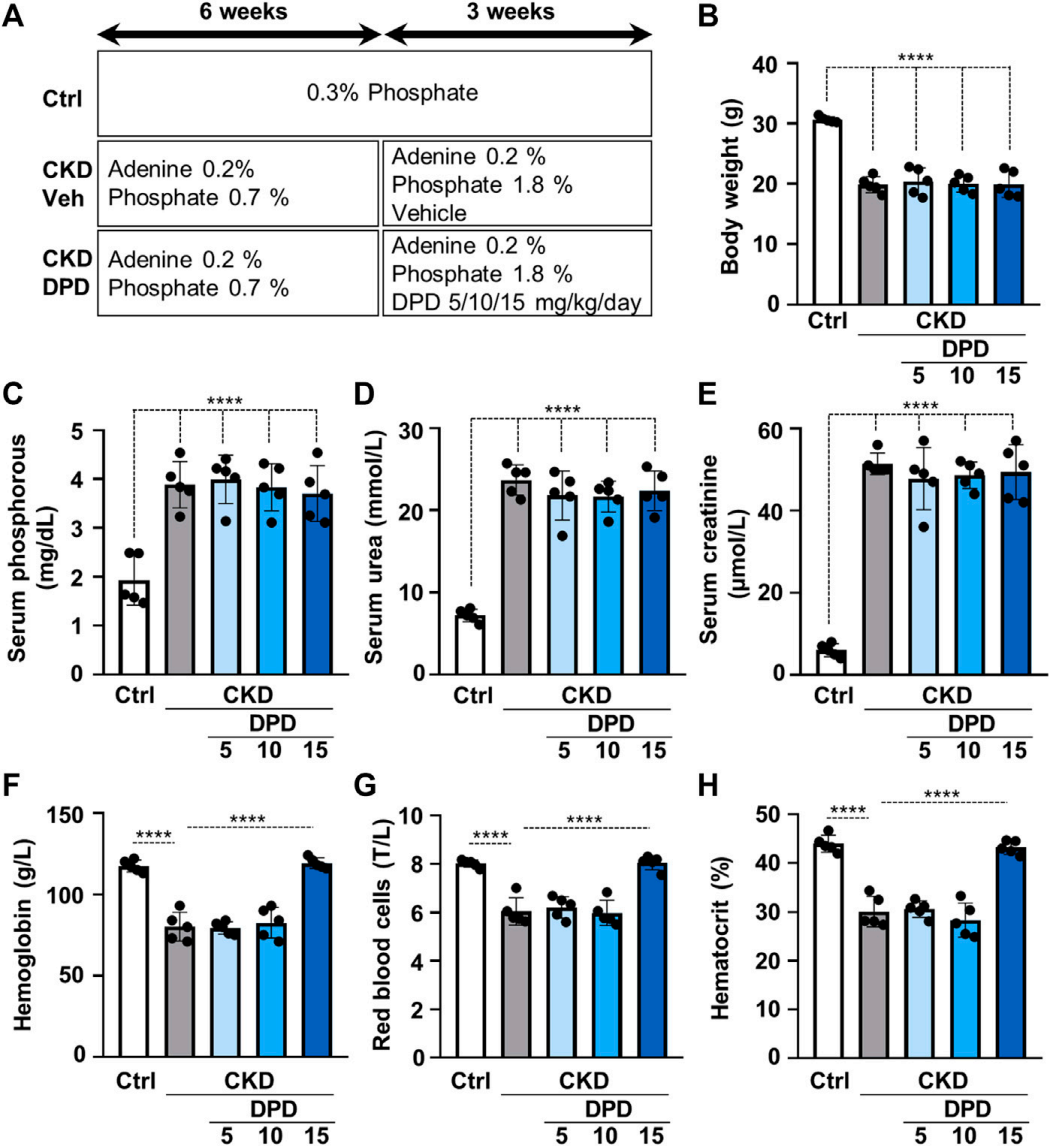
Dose-dependent effect of DPD treatment on renal function and anemia in C57BL/6 mice fed an adenine plus high Pi diet. **(A)** Scheme of the experimental protocol. **(B)** Body weight, **(C)** serum phosphorous, **(D)** serum urea, **(E)** serum creatinine, **(F)** whole blood hemoglobin concentration with **(G)** red blood cell count (T/L), and **(H)** hematocrit values were determined. Data are expressed as mean ± SD, *n* = 5. Ordinary one-way ANOVA followed by Tukey’s multiple comparisons test were used to calculate *p* values. ****p < 0.001.

Then we studied the effect of DPD on anemia and vascular calcification in the murine model of CKD. Mice were fed with a diet containing adenine (0.2%) and elevated phosphate (0.7%) for 6 weeks, and then the phosphate content of the diet was further increased up to 1.8%; mice received this diet for an additional 3 weeks. To determine the efficient dose of DPD that corrects anemia in this particular CKD model, we administered DPD in three different doses: 5, 10, and 15 mg/body weight kg/day orally in the last 3 weeks of the experiment (Figure 4A). The development of CKD with the adenine plus high phosphate diet was associated with significant decrease in body weight (Figure 4B) and increased serum phosphorous, urea, and creatinine levels (Figures 4C–E), regardless of DPD treatment. Parallel to the development of CKD, mice became anemic, and their condition was characterized by reduced Hb concentration, decreased red blood cell count, and low hematocrit levels (Figures 4F–H). Low doses of DPD (5 and 10 mg/kg/day) did not improved anemia, but the highest dose (15 mg/kg/day) efficiently corrected anemia in CKD mice, resulting in normalized Hb concentration, RBC count, and hematocrit levels similar to the controls with normal renal function (Figures 4F–H).

**FIGURE 5:**
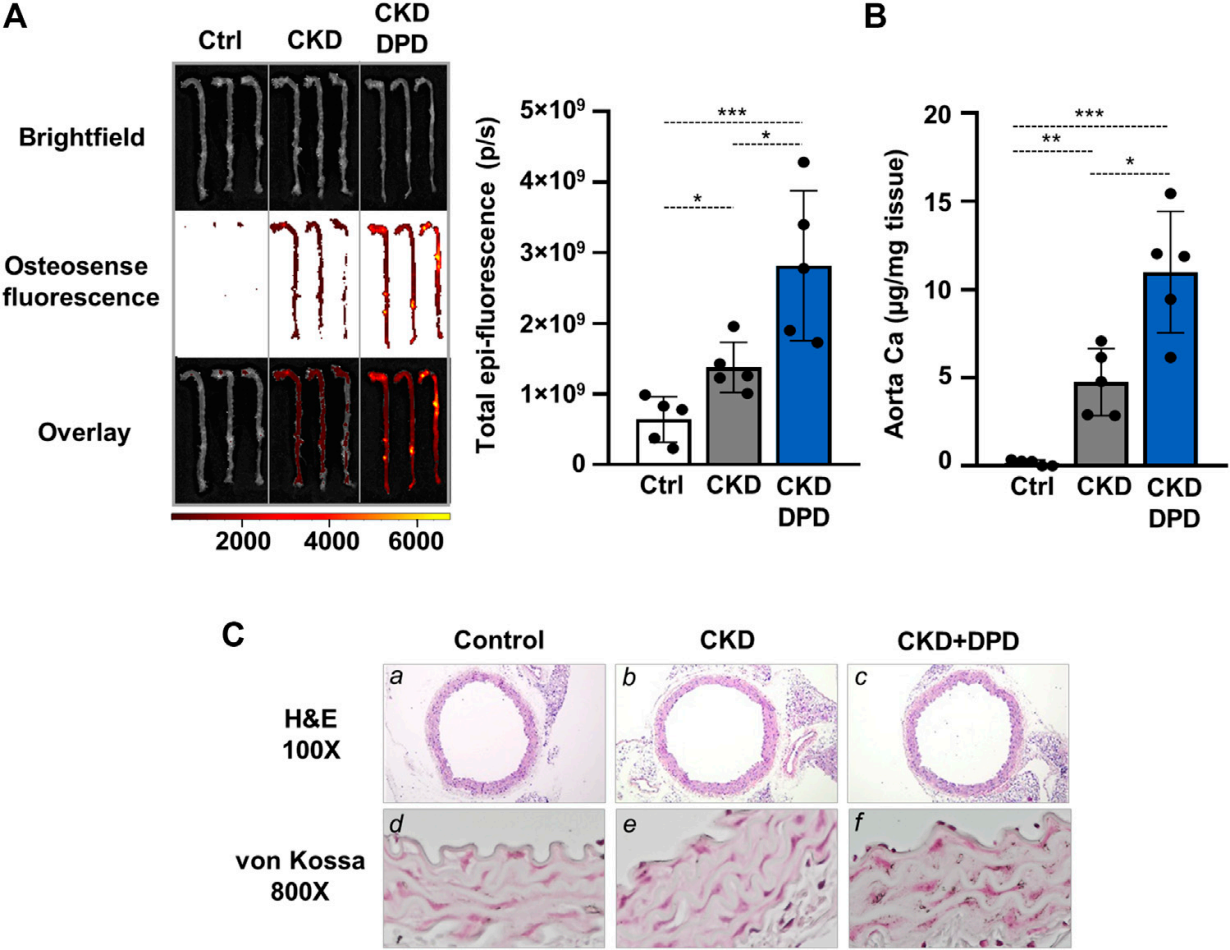
DPD increases aorta calcification in CKD mice with hyperphosphatemia. Mice were treated as detailed in Figure 4A. **(A)** Bright-field and macroscopic fluorescence reflectance imaging of calcification and quantification in control, CKD, and CKD + DPD mice (*n* = 5/group). **(B)** Ca content of aortas derived from control, CKD, and CKD + DPD mice normalized to protein level (*n* = 5/group). **(C)** Histological analysis of aorta obtained from control, CKD, and CKD + DPD mice. Representative H&E and von Kossa-stained aortic sections. Magnification: ×100, ×800. Data are expressed as mean ± SD, *n* = 5. Ordinary one-way ANOVA followed by Tukey’s multiple comparisons test was used to calculate *p* values. *p < 0.05, **p < 0.01, ***p < 0.005.

Next, we also addressed the effect of DPD on aorta calcification *in vivo*. Macroscopic fluorescence reflectance imaging technics was used to investigate the osteogenic activity in whole mouse aortas. Osteosense, a near-infrared fluorescent imaging agent was administered intravenously 24 h before imaging. Fluorescent intensity of the aorta was higher in CKD mice than in control mice with normal renal function (6.41 × 10^8^ ± 3.22 × 10^8^ vs. 1.38 × 10^9^ ± 3.58 × 10^8^ p/s, *p* < 0.05, Figure 5A). Moreover, the osteogenic activity was higher in the aortas derived from DPD-treated CKD mice than in vehicle-treated CKD mice (2.82 × 10^9^ ± 1.06 × 10^9^ vs. 1.38 × 10^9^ ± 3.58 × 10^8^ p/s, *p* < 0.05, Figure 5A). Parallel with this, the Ca level of the aorta was higher in DPD-treated mice than in vehicle-treated CKD mice (4.76 ± 1.89 vs. 10.19 ± 4.97 μg/mg tissue, *p* < 0.05, Figure 5B). von Kossa staining also revealed starting calcification in the media layer of the aorta obtained from DPD-treated CKD mice, whereas calcification was undetectable in vehicle-treated CKD mice and in control mice with normal renal function (Figure 5C).

### HIF-1 Activation Is Critically Involved in DPD-Facilitated Calcification in VSMCs

**FIGURE 6:**
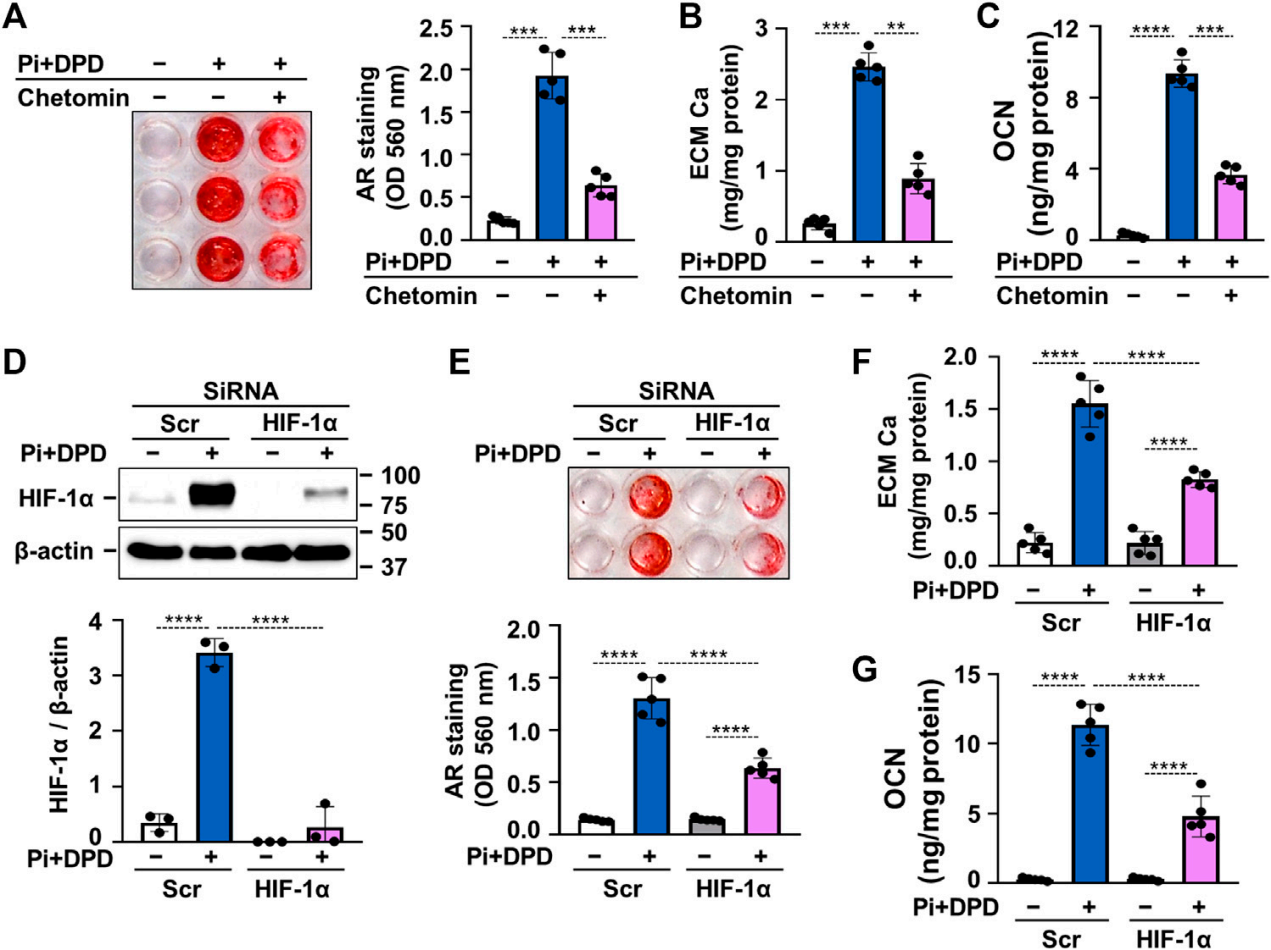
DPD increases calcification of VSMCs through HIF-1 activation. **(A–C)** VSMCs were exposed to Pi (2 mmol/L) and DPD (10 μmol/L) in the presence or absence of HIF-1 inhibitor, chetomin (12.5 nmol/L). **(A)** Representative AR staining (day 4) and quantification. **(B)** Ca content of HCl-solubilized ECM (day 4). **(C)** OCN level in EDTA-solubilized ECM samples (day 6). **(D–G)** VSMCs were exposed to Pi (2 mmol/L) and DPD (10 μmol/L) in the presence of HIF-1α or scrambled siRNA. **(D)** Protein expression of HIF-1α in whole cell lysates (24 h). Membranes were reprobed for β-actin. Representative Western blots and relative expression of HIF-1α normalized to β-actin. **(E)** Representative AR staining (day 4) and quantification. **(F)** Ca content of HCl-solubilized ECM (day 4). **(G)** OCN level in EDTA-solubilized ECM samples (day 6). Data are expressed as mean ± SD, *n* = 5 except **(D)**, where *n* = 3. Ordinary one-way ANOVA followed by Tukey’s multiple comparisons tests were used to calculate *p* values. **p < 0.01, ***p < 0.005, ****p < 0.001.

After establishing that DPD triggers HIF-1 activation in VSMCs, we raised the question whether this mechanism plays a role in the DPD-induced calcification process. To address this, we first used chetomin, a chemical inhibitor of HIF-1 transcriptional activity and investigated the calcification of VSMCs in response to Pi + DPD. As revealed by AR staining, chetomin partially attenuated Pi + DPD-induced calcification of VSMCs (Figure 6A). The inhibitory effect of chetomin on Pi + DPD-induced calcification was confirmed by Ca and OCN measurements from the ECM (Figures 6B,C). Furthermore, siRNA-manipulated knockdown of HIF-1α, the regulatory subunit of the HIF-1 complex, attenuated VSMCs calcification as detected by AR staining, as well as Ca and OCN measurements from the ECM (Figures 6D–G).

### Uric Acid Retention Is Not Involved in the Pro-Calcification Effect of DPD

**FIGURE 7:**
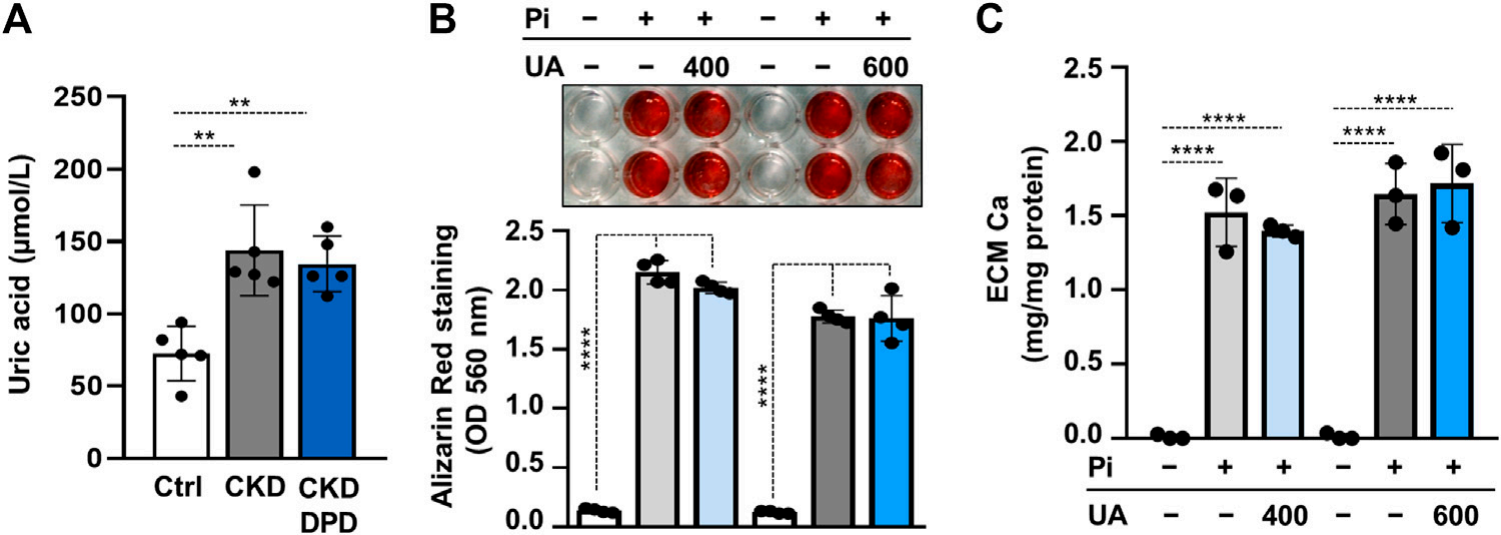
Uric acid retention is not involved in the pro-calcification effect of DPD. **(A)** Serum uric acid levels in control (Ctrl), CKD, and CKD + DPD (15 mg/kg/day) mice (*n* = 5). **(B,C)** VSMCs were exposed to Pi (2 mmol/L) in the presence of uric acid (UA, 400 or 600 μmol/L) or vehicle. **(B)** Representative AR staining (day 4) and quantification (*n* = 4). **(C)** Ca content of HCl-solubilized ECM (day 4, *n* = 3). Data are expressed as mean ± SD. Ordinary one-way ANOVA followed by Tukey’s multiple comparison tests were used to calculate *p* values. **p < 0.01, ****p < 0.001.

DPD is an organic anion which might interfere with transport of other organic anions, specifically uric acid. Uric acid is an important uremic toxin, and recent evidence showed that soluble uric acid promotes atherosclerosis (Kimura et al., 2020). To see whether the pro-calcification effect of DPD relies on uric acid retention, we first determined uric acid levels in mice serum. The induction of CKD and DPD treatment was performed, as shown in Figure 4A. The uric acid level in CKD mice was almost twice as high as the control, whereas its level did not differ between CKD and CKD + DPD (15 mg/kg/day) mice (Figure 7A). Then using an *in vitro* approach, we investigated whether uric acid increases Pi-induced VSMCs calcification. We induced VSMC calcification with Pi (2 mmol/L) in the presence or absence of uric acid (400 and 600 μmol/L). As revealed by AR staining, uric acid did not modify Pi-induced calcification of VSMCs (Figure 7B). This result was confirmed by Ca measurements from the ECM (Figure 7C).

## Discussion

CKD-associated medial calcification is an actively regulated process that involves complex interactions of multiple calcification inducers, inhibitors, and circulating and local factors (Jono et al., 2000; Schoppet et al., 2008; Giachelli, 2009; Paloian and Giachelli, 2014). Transdifferentiation of VSMCs into osteoblast/chondrocyte-like cells is the major cellular mechanism of vascular calcification (Jono et al., 2000; Schoppet et al., 2008; Giachelli, 2009; Paloian and Giachelli, 2014). High Pi is a potent inducer of osteochondrogenic phenotype switch of VSMCs, and it is one of the most relevant inducer of vascular calcification in CKD (Jono et al., 2000; Schoppet et al., 2008; Giachelli, 2009; Paloian and Giachelli, 2014). Studies have shown that hypoxia and HIF-1 signaling are closely associated with kidney disease, and recent evidence proved their implication in vascular calcification (Nangaku and Eckardt, 2007; Gunaratnam and Bonventre, 2009; Mokas et al., 2016; Balogh et al., 2019).

CKD is often accompanied by anemia that requires treatment. DPD is a new generation drug to treat anemia in CKD patients. A recent phase 3 study compared the efficacy and safety of DPD with an ESA (darbepoetin alfa) over 1 year of treatment (Akizawa et al., 2020). That study revealed that oral DPD was generally well tolerated and is non-inferior to ESA in the maintenance of hemoglobin concentration in Japanese dialyzed CKD patients (Akizawa et al., 2020).

DPD is a PHI which acts through the activation of the HIF pathways. Our study provides evidence of a role for DPD in the progression of vascular calcification during CKD. We described synergistic effects between DPD and high Pi during osteochondrogenic differentiation of VSMCs. We report that oral administration of DPD accelerates high Pi-induced calcification in a mouse model of CKD. We also established HIF-1α as major functional contributor of DPD-driven calcification.

Hydroxylation at specific prolyl residues initiates ubiquitination and proteolytic destruction of the regulatory α subunits of HIFs by the ubiquitin/proteasome pathway (Jaakkola et al., 2001). DPD is an inhibitor of prolyl hydroxylases, and here, we show that DPD treatment increases both HIF-1α and HIF-2α expressions in VSMCs (Figure 2). Upon stabilization, HIF α subunits are translocated into the nucleus, heterodimerizes with HIF β subunits, recruits coactivator molecules, that is, p300 and CREB-binding protein, and the complex activates transcription of certain genes controlling cell metabolism and angiogenesis that foster cell survival in a low oxygen environment. Our results revealed that DPD upregulates Glut-1, an important target gene of HIF, proving that DPD potently activated the HIF pathway in human VSMCs (Figure 2).

Growing evidence suggests that diseases with hypoxemia and/or hypoxia, such as asthma, chronic obstructive pulmonary disease, and obstructive sleep apnea are associated with increased vascular calcification (Green et al., 2006; Williams et al., 2014; Tachikawa et al., 2015). Moreover, Mokas et al. showed that hypoxia synergizes with high Pi to enhance osteochondrogenic transdifferentiation of VSMCs (Mokas et al., 2016). Furthermore, we reported recently that hypoxia itself is a pro-calcifying factor and is able to induce osteochondrogenic transdifferentiation and ECM calcification of VSMCs (Balogh et al., 2019). These observations warranted us to test the pro-calcifying potential of DPD.

We chose a cellular model of vascular calcification in which we induced calcification of VSMCs with high Pi because DPD is a drug intended to be used in progressive CKD patients who develop positive phosphate balance. Here, we reported that similar to hypoxia, DPD intensifies high Pi-induced osteochondrogenic transdifferentiation, ECM calcification of VSMCs *in vitro*, and aorta calcification *ex vivo* (Figure 3). Similar results were obtained in previous studies with another PHI, roxadustat (FG-4592), that also enhanced VSMCs calcification under high phosphate conditions (Mokas et al., 2016; Nagy et al., 2020).

Several phase 3 and phase 2 studies demonstrated that DPD is effective in improving hemoglobin levels of CKD patients (Ishii et al., 2021). These clinical trials have not reported serious adverse events or obvious off-target effects of DPD (Li et al., 2018; Ishii et al., 2021). In 2020, DPD was approved for the treatment of patients with CKD-associated anemia in Japan (Dhillon, 2020).

After seeing the pro-calcifying action of DPD in elevated phosphate condition *in vitro*, we aimed to test the effect of DPD on anemia and calcification *in vivo*. In order to do this, we applied an adenine-induced CKD mice model, in which high-phosphate condition was approached by a diet rich in phosphorous. We tested three doses of DPD (5, 10, and 15 mg/kg/day) and found that DPD at the dose of 15 mg/kg/day corrected anemia of CKD mice completely, whereas the lower doses did not improve the hematological parameters (Figure 4). This dose is higher than the dose reported earlier by Ariazi et al. who tested the effect of DPD (3, 10, and 30 mg/kg/day) on the Hb level and reticulocyte number in normal female B6D2F1 mice during the preclinical characterization of DPD (Ariazi et al., 2017). They found that a daily administration of 3 mg/kg DPD increased the reticulocyte number and hemoglobin concentration significantly. There could be several reasons for this discrepancy, such as the mice model (CKD vs. healthy), the initial anemia status (moderate to severe anemia vs. non-anemia), the gender (male vs. female), or the genetic background (C57BL/6 vs. B6D2F1). Here, we reported that besides its beneficial effect in correcting anemia, DPD at the dose of 15 mg/kg/day accelerated aorta calcification in CKD mice with high plasma phosphate levels (Figure 5). We showed that DPD stabilized both HIF-1α and HIF-2α in VSMCs. Chetomin that blocks the interaction of HIF α subunits with transcriptional co-activators, thereby attenuating hypoxia-inducible transcription, inhibited calcification triggered by DPD + Pi (Figure 6). Both the ubiquitously expressed HIF-1α and the more cell-specific HIF-2α are important regulators of the hypoxia response (Loboda et al., 2010). Although both HIF-1α and HIF-2α subunits heterodimerize with the HIF-1β subunit in the nucleus, and the HIF1 and HIF2 bind to the same hypoxia responsive elements of target genes, their effect on the expression of some genes may be specific (Loboda et al., 2010). There is a consensus that the PHIs increase EPO expression mainly through HIF-2. On the other hand, previous reports provided evidence that sustained HIF-1α stabilization induces VSMC calcification in both normal and high phosphate conditions (Mokas et al., 2016; Balogh et al., 2019). Therefore, focusing on HIF-1α, here, we showed that the pro-calcifying effect of DPD is dependent on HIF-1α stabilization (Figure 6).

Uremic toxins accumulate in CKD patient’s plasma and contribute to the pathology of the disease. One example is uric acid, an end-product of purine metabolism that normally excreted through the urine (Kumagai et al., 2017). Excess uric acid can precipitate causing gout, whereas soluble uric acid promotes atherosclerosis and further exacerbates CKD (Kumagai et al., 2017; Kimura et al., 2020). There are conflicting results about the association between uric acid levels and vascular calcification (Neogi et al., 2011; Malik et al., 2016; Yan et al., 2019). Here, we tested the hypothesis that DPD interferes with urinary excretion of uric acid, and uric acid increases VSMCs calcification. Our results showed that uric acid levels were similar in DPD-treated and non-treated CKD mice, and uric acid does not influence Pi-induced VSMCs calcification (Figure 7).

To our knowledge, this is the first study that addressed the potential pro-calcifying effect of DPD. We found that DPD treatment accelerates phosphate-induced vascular calcification *in vitro* in primary VSMCs, *ex vivo* in mouse aorta rings, and *in vivo* in a murine CKD model with a high plasma phosphorous level. We assumed that administration of DPD in CKD patients with hyperphosphatemia could increase the risk of vascular calcification. Further investigation with an extended follow-up period is warranted to evaluate the possible risks of sustained HIF elevation by DPD in accelerating calcification in CKD patients with hyperphosphatemia.

## Data Availability

The raw data supporting the conclusion of this article will be made available by the authors, without undue reservation.

## Abbreviations

AR, alizarin red; CKD, chronic kidney disease; Ctrl, control; DMEM, Dublecco’s modified eagle medium; DMSO, dimethyl sulphoxide; DPBS, Dulbecco’s phosphate-buffered saline; DPD, daprodustat; ECM, extracellular matrix; EPO, erythropoietin; ER, endoplasmic reticulum; ESAs, erythropoiesis-stimulating agents; FBS, fetal bovine serum; FDA, U.S. Food and Drug Administration; GM, growth medium; H&E, hematoxylin eosin; HIF-1, hypoxia inducible factor 1; MCV, mean cell volume; OCN, osteocalcin; OD, optical density; OM, osteogenic medium; PHD, prolyl hydroxylase domain-containing; PHI, prolyl hydroxylase inhibitor; Pi, inorganic phosphate; RBC, red blood cell; VEGF-A, vascular endothelial growth factor A; VSMCs, vascular smooth muscle cells.

## References

[B1] Akizawa T. Nangaku M. Yonekawa T. Okuda N. Kawamatsu S. Onoue T. (2020). Efficacy and Safety of Daprodustat Compared with Darbepoetin Alfa in Japanese Hemodialysis Patients with Anemia: A Randomized, Double-Blind, Phase 3 Trial. Clin. J. Am. Soc. Nephrol. 15, 1155–1165. 10.2215/CJN.16011219 32723804 PMC7409739

[B2] Ariazi J. L. Duffy K. J. Adams D. F. Fitch D. M. Luo L. Pappalardi M. (2017). Discovery and Preclinical Characterization of GSK1278863 (Daprodustat), a Small Molecule Hypoxia Inducible Factor-Prolyl Hydroxylase Inhibitor for Anemia. J. Pharmacol. Exp. Ther. 363, 336–347. 10.1124/JPET.117.242503 28928122

[B3] Babitt J. L. Lin H. Y. (2012). Mechanisms of Anemia in CKD. J. Am. Soc. Nephrol. 23, 1631–1634. 10.1681/ASN.2011111078 22935483 PMC3458456

[B4] Balogh E. Tóth A. Méhes G. Trencsényi G. Paragh G. Jeney V. (2019). Hypoxia Triggers Osteochondrogenic Differentiation of Vascular Smooth Muscle Cells in an HIF-1 (Hypoxia-Inducible Factor 1)-Dependent and Reactive Oxygen Species-dependent Manner. Arterioscler Thromb. Vasc. Biol. 39, 1088–1099. 10.1161/ATVBAHA.119.312509 31070451

[B5] Batchelor E. K. Kapitsinou P. Pergola P. E. Kovesdy C. P. Jalal D. I. (2020). Iron Deficiency in Chronic Kidney Disease: Updates on Pathophysiology, Diagnosis, and Treatment. J. Am. Soc. Nephrol. 31, 456–468. 10.1681/ASN.2019020213 32041774 PMC7062209

[B6] Dhillon S. (2020). Daprodustat: First Approval. Drugs 80, 1491–1497. 10.1007/s40265-020-01384-y 32880805 PMC7471535

[B7] Di Angelantonio E. Danesh J. Eiriksdottir G. Gudnason V. (2007). Renal Function and Risk of Coronary Heart Disease in General Populations: New Prospective Study and Systematic Review. Plos Med. 4, e270. 10.1371/journal.pmed.0040270 17803353 PMC1961630

[B8] Eschbach J. W. Egrie J. C. Downing M. R. Browne J. K. Adamson J. W. (1987). Correction of the Anemia of End-Stage Renal Disease with Recombinant Human Erythropoietin. Results of a Combined Phase I and II Clinical Trial. N. Engl. J. Med. 316, 73–78. 10.1056/NEJM198701083160203 3537801

[B9] Gafter-Gvili A. Schechter A. Rozen-Zvi B. (2019). Iron Deficiency Anemia in Chronic Kidney Disease. Acta Haematol. 142, 44–50. 10.1159/000496492 30970355

[B10] Giachelli C. M. (2009). The Emerging Role of Phosphate in Vascular Calcification. Kidney Int. 75, 890–897. 10.1038/ki.2008.644 19145240 PMC2807740

[B11] Green F. H. Butt J. C. James A. L. Carroll N. G. (2006). Abnormalities of the Bronchial Arteries in Asthma. Chest 130, 1025–1033. 10.1378/chest.130.4.1025 17035434

[B12] Gunaratnam L. Bonventre J. V. (2009). HIF in Kidney Disease and Development. J. Am. Soc. Nephrol. 20, 1877–1887. 10.1681/ASN.2008070804 19118148

[B13] Hanna R. M. Streja E. Kalantar-Zadeh K. (2021). Burden of Anemia in Chronic Kidney Disease: Beyond Erythropoietin. Adv. Ther. 38, 52–75. 10.1007/s12325-020-01524-6 PMC7854472 33123967

[B14] Ishii T. Tanaka T. Nangaku M. (2021). Profile of Daprodustat in the Treatment of Renal Anemia Due to Chronic Kidney Disease. Tcrm Vol. 17, 155–163. 10.2147/TCRM.S293879 PMC7898206 33628028

[B15] Jaakkola P. Mole D. R. Tian Y. M. Wilson M. I. Gielbert J. Gaskell S. J. (2001). Targeting of HIF-alpha to the von Hippel-Lindau ubiquitylation complex by O2-regulated prolyl hydroxylation. Science 292, 468–472. 10.1126/science.1059796 11292861

[B16] Jono S. McKee M. D. Murry C. E. Shioi A. Nishizawa Y. Mori K. (2000). Phosphate Regulation of Vascular Smooth Muscle Cell Calcification. Circ. Res. 87. 10.1161/01.res.87.7.e10 11009570

[B17] Kimura Y. Yanagida T. Onda A. Tsukui D. Hosoyamada M. Kono H. (2020). Soluble Uric Acid Promotes Atherosclerosis via AMPK (AMP-Activated Protein Kinase)-Mediated Inflammation. Arterioscler. Thromb. Vasc. Biol. 40, 570–582. 10.1161/ATVBAHA.119.313224 31996020

[B18] Koulouridis I. Alfayez M. Trikalinos T. A. Balk E. M. Jaber B. L. (2013). Dose of Erythropoiesis-Stimulating Agents and Adverse Outcomes in CKD: A Metaregression Analysis. Am. J. Kidney Dis. 61, 44–56. 10.1053/j.ajkd.2012.07.014 22921639 PMC3525813

[B19] Kumagai T. Ota T. Tamura Y. Chang W. X. Shibata S. Uchida S. (2017). Time to Target Uric Acid to Retard CKD Progression. Clin. Exp. Nephrol. 21, 182–192. 10.1007/S10157-016-1288-2 27339448

[B20] Li W. Zhao Y. Fu P. (2018). Hypoxia Induced Factor in Chronic Kidney Disease: Friend or Foe. Front. Med. (Lausanne) 4, 259. 10.3389/fmed.2017.00259 29404328 PMC5786558

[B21] Loboda A. Jozkowicz A. Dulak J. (2010). HIF-1 and HIF-2 Transcription Factors-Ssimilar but Not Identical. Mol. Cell 29, 435–442. 10.1007/S10059-010-0067-2 20396958

[B22] Locatelli F. Bárány P. Covic A. De Francisco A. Del Vecchio L. Goldsmith D. (2013). Kidney Disease: Improving Global Outcomes Guidelines on Anaemia Management in Chronic Kidney Disease: A European Renal Best Practice Position Statement. Nephrol. Dial. Transpl. 28, 1346–1359. 10.1093/ndt/gft033 23585588

[B23] Locatelli F. Fishbane S. Block G. A. MacDougall I. C. (2017). Targeting Hypoxia-Inducible Factors for the Treatment of Anemia in Chronic Kidney Disease Patients. Am. J. Nephrol. 45, 187–199. 10.1159/000455166 28118622

[B24] Malhotra R. Mauer A. C. Lino Cardenas C. L. Guo X. Yao J. Zhang X. (2019). HDAC9 Is Implicated in Atherosclerotic Aortic Calcification and Affects Vascular Smooth Muscle Cell Phenotype. Nat. Genet. 51, 1580–1587. 10.1038/s41588-019-0514-8 31659325 PMC6858575

[B25] Malik R. Aneni E. C. Shahrayar S. Freitas W. M. Ali S. S. Veledar E. (2016). Elevated Serum Uric Acid Is Associated with Vascular Inflammation but Not Coronary Artery Calcification in the Healthy Octogenarians: the Brazilian Study on Healthy Aging. Aging Clin. Exp. Res. 28, 359–362. 10.1007/S40520-015-0395-3 26084248

[B26] Martin K. J. González E. A. (2007). Metabolic Bone Disease in Chronic Kidney Disease. J. Am. Soc. Nephrol. 18, 875–885. 10.1681/ASN.2006070771 17251386

[B27] Maxwell P. H. Eckardt K. U. (2016). HIF Prolyl Hydroxylase Inhibitors for the Treatment of Renal Anaemia and beyond. Nat. Rev. Nephrol. 12, 157–168. 10.1038/nrneph.2015.193 26656456

[B28] McCullough P. A. Barnhart H. X. Inrig J. K. Reddan D. Sapp S. Patel U. D. (2013). Cardiovascular Toxicity of Epoetin-Alfa in Patients with Chronic Kidney Disease. Am. J. Nephrol. 37, 549–558. 10.1159/000351175 23735819

[B29] Metzen E. Ratcliffe P. J. (2004). HIF Hydroxylation and Cellular Oxygen Sensing. Biol. Chem. 385, 223–230. 10.1515/BC.2004.016 15134335

[B30] Mokas S. Larivière R. Lamalice L. Gobeil S. Cornfield D. N. Agharazii M. (2016). Hypoxia-inducible Factor-1 Plays a Role in Phosphate-Induced Vascular Smooth Muscle Cell Calcification. Kidney Int. 90, 598–609. 10.1016/j.kint.2016.05.020 27470678

[B31] Nagy A. Pethő D. Gáll T. Zavaczki E. Nyitrai M. Posta J. (2020). Zinc Inhibits HIF-Prolyl Hydroxylase Inhibitor-Aggravated VSMC Calcification Induced by High Phosphate. Front. Physiol. 10, 1584. 10.3389/FPHYS.2019.01584 32009983 PMC6974455

[B32] Nangaku M. Eckardt K. U. (2007). Hypoxia and the HIF System in Kidney Disease. J. Mol. Med. (Berl) 85, 1325–1330. 10.1007/s00109-007-0278-y 18026918

[B33] Neogi T. Terkeltaub R. Ellison R. C. Hunt S. Zhang Y. (2011). Serum Urate Is Not Associated with Coronary Artery Calcification: the NHLBI Family Heart Study. J. Rheumatol. 38, 111–117. 10.3899/JRHEUM.100639 20889594 PMC3119360

[B34] Palmer S. C. Navaneethan S. D. Craig J. C. Johnson D. W. Tonelli M. Garg A. X. (2010). Meta-analysis: Erythropoiesis-Stimulating Agents in Patients with Chronic Kidney Disease. Ann. Intern. Med. 153, 23–33. 10.7326/0003-4819-153-1-201007060-00252 20439566

[B35] Paloian N. J. Giachelli C. M. (2014). A Current Understanding of Vascular Calcification in CKD. Am. J. Physiol. Ren. Physiol 307, F891–F900. 10.1152/ajprenal.00163.2014 PMC4200295 25143458

[B36] Robles N. R. (2016). The Safety of Erythropoiesis-Stimulating Agents for the Treatment of Anemia Resulting from Chronic Kidney Disease. Clin. Drug Investig. 36, 421–431. 10.1007/s40261-016-0378-y 26894799

[B37] Sarnak M. J. Levey A. S. Schoolwerth A. C. Coresh J. Culleton B. Hamm L. L. (2003). Kidney Disease as a Risk Factor for Development of Cardiovascular Disease: A Statement from the American Heart Association Councils on Kidney in Cardiovascular Disease, High Blood Pressure Research, Clinical Cardiology, and Epidemiology and Prevention. Circulation 108, 2154–2169. 10.1161/01.CIR.0000095676.90936.80 14581387

[B38] Schoppet M. Shroff R. C. Hofbauer L. C. Shanahan C. M. (2008). Exploring the Biology of Vascular Calcification in Chronic Kidney Disease: What's Circulating. Kidney Int. 73, 384–390. 10.1038/sj.ki.5002696 18046319

[B39] Semenza G. L. (2001). HIF-1, O(2), and the 3 PHDs: How Animal Cells Signal Hypoxia to the Nucleus. Cell 107, 1–3. 10.1016/S0092-8674(01)00518-9 11595178

[B40] Semenza G. L. Wang G. L. (1992). A Nuclear Factor Induced by Hypoxia via De Novo Protein Synthesis Binds to the Human Erythropoietin Gene Enhancer at a Site Required for Transcriptional Activation. Mol. Cel. Biol. 12, 5447–5454. 10.1128/mcb.12.12.5447 PMC360482 1448077

[B41] Tachikawa R. Koyasu S. Matsumoto T. Hamada S. Azuma M. Murase K. (2015). Obstructive Sleep Apnea and Abdominal Aortic Calcification: Is There An association Independent of Comorbid Risk Factors. Atherosclerosis 241, 6–11. 10.1016/j.atherosclerosis.2015.04.801 25935114

[B42] Tani T. Orimo H. Shimizu A. Tsuruoka S. (2017). Development of a Novel Chronic Kidney Disease Mouse Model to Evaluate the Progression of Hyperphosphatemia and Associated mineral Bone Disease. Sci. Rep. 7, 2233. 10.1038/s41598-017-02351-6 28533541 PMC5440375

[B43] Webster A. C. Nagler E. V. Morton R. L. Masson P. (2017). Chronic Kidney Disease. Lancet 389, 1238–1252. 10.1016/S0140-6736(16)32064-5 27887750

[B44] Williams M. C. Murchison J. T. Edwards L. D. Agustí A. Bakke P. Calverley P. M. (2014). Coronary Artery Calcification Is Increased in Patients with COPD and Associated with Increased Morbidity and Mortality. Thorax 69, 718–723. 10.1136/thoraxjnl-2012-203151 24473329

[B45] Yan B. Liu D. Zhu J. Pang X. (2019). The Effects of Hyperuricemia on the Differentiation and Proliferation of Osteoblasts and Vascular Smooth Muscle Cells Are Implicated in the Elevated Risk of Osteopenia and Vascular Calcification in Gout: An *In Vivo* and *In Vitro* Analysis. J. Cel. Biochem. 120, 19660–19672. 10.1002/JCB.29272 31407397

